# Analysis of the mental health service provision in Qatar: Progressing in community mental health in a Middle East country

**DOI:** 10.3389/fpsyt.2022.1095788

**Published:** 2022-12-16

**Authors:** Jose A. Salinas-Perez, Suhaila Ghuloum, Mencia R. Gutierrez-Colosia, Nasser Bagheri, Luis Salvador-Carulla

**Affiliations:** ^1^Department of Quantitative Methods, Universidad Loyola Andalucía, Dos Hermanas, Seville, Spain; ^2^Mental Health Policy Unit, Faculty of Health, Health Research Institute, University of Canberra, Canberra, ACT, Australia; ^3^Mental Health Services, Hamad Medical Corporation, Doha, Qatar; ^4^Department of Psychology, Universidad Loyola Andalucía, Dos Hermanas, Seville, Spain; ^5^Menzies Centre for Health Policy and Economics, School of Public Health, The University of Sydney, Sydney, NSW, Australia

**Keywords:** mental health service, integrated Atlas, DESDE-LTC, healthcare ecosystem, health planning, Qatar

## Abstract

**Background:**

Qatari health planning in the last decade aimed to make the transition from the traditional hospital-based psychiatric care to a community-based care, building an integrated and comprehensive mental health system. The objective of this study was to explore the mental health service provision in Qatar in 2018 and 2022. This time span coincided with two mental health plans (2013–2018 and 2019–2022) and one health plan (2018–2022).

**Methods:**

This study followed a healthcare ecosystem approach, including context analysis and the standard description and classification of mental health services. Service provision was studied applying DESDE-LTC system (Description and Evaluation of Services and DirectoriEs–Long Term Care), an internationally validated methodology to assess and describe mental health services. Service data were analyzed along with sociodemographic indicators from public statistics to know the care context.

**Results:**

The availability of specialized mental health services increased for adults, although it remained the same for other age groups. The diversity of care and the weight of health-related care over social-related care also remained quite similar. It was noteworthy the development of new services for young adults, migrant workers, and female populations.

**Conclusion:**

This was the first time that this service research methodology has been applied in a Middle East country to study its mental healthcare pattern. The analysis of the mental healthcare pattern in the study time period showed the continued progress toward community-based care in Qatar in the framework of three health plans and despite the unexpected COVID-19 world pandemic.

## 1 Introduction

Qatar has experienced a great development of its healthcare system accompanying its massive economic growth in the last two decades. In this framework, the Qatar’s National Mental Health Strategy (2013–2018) (1), the National Mental Health and Wellbeing Strategic Framework 2019–2022 (2), and the National Health Strategy (2018–2022) (3) aimed to make the transition from the traditional hospital-based psychiatric care to a community-based care, building an integrated and comprehensive mental health system. Furthermore, in 2016 a Law on Rights of Patients with Mental Illness was adopted to regulate the care, treatment and rights of patients with mental disorders (4), but it has not been implemented yet (5). This set of laws and regulations, along with previous policies (6), place Qatar at the front of mental health planning in the WHO Eastern Mediterranean Region (EMRO).

Hamad Medical Corporation (HMC) is the public provider of specialized healthcare in Qatar, including mental healthcare. The other key piece of the Qatari healthcare system is the Primary Health Care Corporation (PHCC). Both organizations work together to develop an integrated network of services to manage mental disorders from the primary care in a stepped care approach, and to refer cases, when needed, to the specialized care. Public mental health system is provided free of charge to all Qatari citizens. Non-national population must pay 20% of the cost of medications in the outpatient setting, while inpatient care is free. Additionally, Sidra Medicine, a non-profit organization, provides mental healthcare for children and women in the perinatal period, up to a year post delivery. Recently, the Qatari mental health system has been assessed as entirely efficient in a comparison with other Middle East countries (7).

However, some challenges raised in the literature (5, 8, 9) include: insufficient training and lack of motivation of the primary care workforce; limited availability of mental health services and professionals, especially psychologists, nurses, and social workers, and a high degree of stigma that limits mental healthcare seeking behavior. Delays and organizational challenges are associated with implementing the mental health law. Furthermore, clinical services use Western-based treatment approaches and assessment scales that need to be adapted to the local context. Moreover, the migrant population (over 75% of the total population) presents its own challenges with limited access to mental healthcare.

The development of the Qatari community mental health system since the 70’s (10), with the coexistence of previous institutionalized services (in a transformation process now), and new services, has hampered a comprehensive study of its mental health service provision to verify if it is coherent with the principles of the last health plans. The WHO’s Mental Health Atlas 2020 provided service availability information (11), but it was collected following a top-down perspective and its comparability is limited. Thus, this study requires to identify homogenous units of analysis to assure the commensurability (comparing like-with-like), and a classification of services based on their actual performance to avoid problems of terminological variability.

This study aims to explore the mental health service provision in Qatar, which is shifting its psychiatric healthcare toward a non-hospital-based community model, in the years 2018 and 2022. This time span coincides with two mental health plans and one health plan.

## 2 Materials and methods

### 2.1 Design

This study followed a healthcare ecosystem approach (12, 13), which follows a bottom-up perspective that includes the context analysis and the standard description and classification of mental health services.

### 2.2 Standard service description and classification

Description and Evaluation of Services and DirectoriEs–Long Term Care (DESDE-LTC) (14) is an international validated tool for the standard classification of services in multiple sectors (health, social, justice, education, etc.). DESDE-LTC has been used in studies around the world (15). It includes a coding system organized in tree or diagram according to the care provided (Residential, Day, Outpatient, Accessibility, Information, or Self-help). These main care typologies are subdivided in several sub-levels to reflect aspects like severity of the user, frequency of care, or length of stay, among other characteristics.

The DESDE-LTC system uses a unit of analysis called “Basic Stable Input of Care” (BSIC). BSICs are defined as stable teams of professionals dedicated to providing care to a population group of health consumers. Stability is both temporal (at least 3 years old) and organizational (has its own facility, the same team of professionals, administrative or accounting support and an independent budget). For instance, an acute psychiatric unit in a general hospital (with its own premises and administration) that has a team of professionals delivering care to a group of users. BSICs are described using a Main Type of Care (MTC), which is a code defined by the main activity of the service instead of its name. Following the previous example, the main function of this acute psychiatric unit (BSIC) would be to provide acute residential care to inpatients (first MTC code). Sometimes, a BSIC performs an additional main activity that needs a secondary MTC. In this case, the acute psychiatric unit could provide emergency care for outpatients (second MTC code).

In this study services had to fulfill the following inclusion criteria: (a) be specialized mental health services; (b) target any age group; (c) have universal access or not having a significant out-of-pocket cost; (d) have a temporal stability of at least 3 years; (e) have its own administrative support, space, finances, and documentation; (f) deliver care to the study area; and (g) provide direct care or support to consumers. Services targeted for alcohol and other substance use disorders were excluded.

### 2.3 Data collection and analysis

The Qatari mental healthcare ecosystem is described through a set of demographic and socioeconomic measures drawn from the Psicost mental health indicators set (16). Secondary data were collected from the Qatari statistical agency (17), the World Bank (18), and the WHO Mental Health Atlas (11). Service information was collected through face-to-face interviews conducted by trained researchers in 2018 and 2022 with a questionnaire based on the service inventory of DESDE-LTC.

The mental health service provision is measured in raw figures and in availability rates per 100,000 inhabitants according to three target age groups (children and adolescents, adults, and older adults). The diversity of care is the number of different DESDE-LTC codes used to describe the mental healthcare. Finally, the balance of care provides the share between health-related and social care according to the codes described.

## 3 Results

### 3.1 Qatar population context

As the Qatari territory is desert, the population live mainly in urban centers, especially in Doha where over the 90% of the population resides. The demographic characteristics of Qatar (Table 1) are strongly influenced by the significant increase of the migrant population in the last few decades due to the dramatic economic growth. Over 75% of the 2.8 million inhabitants are foreign workers, mainly from India, Bangladesh, and Nepal. Foreign workers are mostly adult men, residing alone in the country, working in the construction sector. Subsequently, women proportion is below 50%, and the dependency and aging indexes are very low.

**TABLE 1:** Sociodemographic description of Qatar.

Indicators	Values
**Demographics**	
Inhabitants (2020)^1^	2,833,679
Population density (2020)^1^ (inhabitants/squared kilometers)	231.70
Dependency index (2020)^1^ (population aged under 15 years and over 64 years/population aged between 15 and 64 years × 100)	20.49
Aging index (2020)^1^ (population aged over 64 years/population aged under 15 years × 100)	7.73
Sex index (2020)^1^ (females/males × 100)	39.89
Non-married rate (2020)^1^ (never married, widowed and divorced people/population aged over 14 years × 100)	33.00
International migrant stock (2015)^2^ (people born abroad/total inhabitants × 100)	75.5
**Economics**	
GDP per capita (2020)^2^ (US$ PPP)	89, 961.4
Unemployment rate (2020)^1^ (unemployed population/economically active population × 100)	0.10
**Health**	
Disability rate (2020)^1^ (individuals with disabilities/total population × 1,000)	1.19
Death rate (2020)^1^ (registered deaths/total population × 1,000)	0.77
Psychiatric inpatient admissions (2020)^3^ (inpatient admissions/population × 100,000)	52.79
Psychiatric discharges rate (Hamad Medical Corporation) (2019)^1^ (F01-F99 discharges/total population × 1,000)	0.86
Average daily psychiatric stay (Hamad Medical Corporation) (2019)^1^ (F01-F99 daily stays)	8.20
Psychiatric outpatient visits (2020)^3^ (Outpatient visits/population × 100,000)	3, 469.76
Mental disorder prevalence (2009)^4^ (people with mental illness/total population × 100)	36.6

^1^Planning and Statistics Authority–Government of Qatar (17); ^2^World Bank (18); ^3^WHO’s Mental Health Atlas (11); and ^4^Ghuloum et al. (19).

The Qatari economy is based on the trade of oil and is among the countries with higher gross domestic product (GDP) per capita. Moreover, the unemployment is almost inexistent. However, the income is not equally distributed between the Qatari people and migrants, the latter living in poorer conditions.

The prevalence of mental disorder was estimated at 36.6% of the adult population in the unique study on prevalence carried out in Qatar (19). Depression and anxiety were the most frequent disorders, holding women at a higher risk.

### 3.2 Mental healthcare

Although the Qatari mental health system intends to base on stepped care approach in the next few years, currently the usual gate to the system is the specialized services, either as self-referrals or through other secondary or tertiary health providers. Primary care professionals are being trained to treat mild cases of depression and anxiety, and guidelines as to when to refer to the psychiatry facilities are available. However, primary care is still far from becoming the gatekeeper of the mental healthcare.

The specialized network was composed of community-based services, such as outreach services, day-care, community residences, and hospital acute and long-stay wards. In 2018, the Qatari mental health service system was composed of 20 BSIC that were described through 21 MTC (codes). The description used nine different codes to define the diversity of care. Regarding the balance of care, most of MTC were healthcare-related (90.5%), while only the 9.5% of codes were not health related.

**FIGURE 1:**
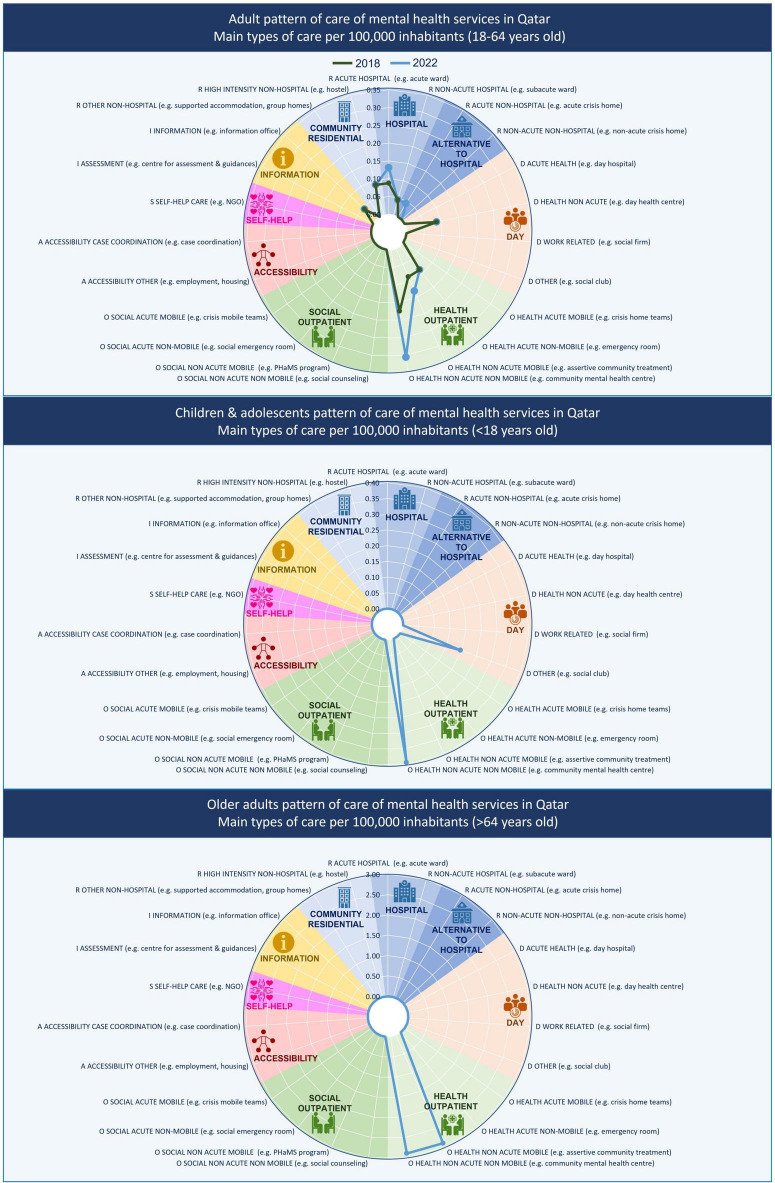
Mental health service pattern by age group in Qatar (2018–2022).

Overall, five services delivered residential care. Two acute care units in the psychiatry hospital (DESDE-LTC code R2) and a long-term hospital unit (code R4) at Rumailah hospital in Doha; and two community residences with high support (24 h) (code R11) in a community mental health facility in Al-Rayyan near Doha. The availability rate of acute hospital care and high intensity non-hospital care coincided in 0.10 per 100,000 inhabitants (Figure 1). The residential services were exclusively targeted for males or females. Children and adolescents and older people did not have specific residential services. Older adults were treated in general adult services, while children and adolescents with a psychiatric issue were admitted in the pediatric unit of the general hospital and more often also in the general adult services. There were 65 beds in the acute units (2.84 beds per 100,000 inhabitants) and 15 in the community residences (0.65 bed per 100,000).

Day-care was described with three codes. Two codes for adults’ health related care (males and females), where the users attended once or twice per week (D8.1), and a summer camp for children and adolescents providing non-health-related care (D4.3). All of them were located in the community-based facility in Al-Rayyan. The availability of the adult health-related day care was 0.1, and the infant social day care was 0.05 per 100,000 inhabitants.

The services described as delivering outpatient care were the most numerous. All of them provided health-related non-acute outpatient care. There were two outreach services for adults (males and females), and one for older people (both sexes) (O7.1); and two liaison services (O4.1). Moreover, there were seven services delivering non-mobile care: four for adults (codes O9.1), two for children and adolescents (O9.1), and one for older people (O10.1). The availability rate for mobile non-acute outpatient care was 0.09 per 100,000 inhabitants for adults and 2.9 for older adults. The non-mobile outpatient care had an availability of 0.26 for adults, 0.39 for children and adolescents, and 2.9 for older people.

Additionally, an Non-Governmental Organization (NGO) provided online psychological counseling (I2.1.2) with an availability rate of 0.04 per 100,000 inhabitants.

In 2022, this study identified 26 BSIC that were described through 27 MTC. New services were created and most of the existing services remained. Only the NGO, delivering information for care, and the former subacute unit in the Rumailah hospital ceased to operate. New services included non-mobile outpatient care, such as a university counseling center (O8.1), and a couple of psychiatry consultation in a healthcare center (O10.1), and a general hospital (O9.1) focused on providing care for the lower-skilled migrant workers through culturally competent workforce. Mobile outpatient care was complemented with a new outreach service (O7.1) at the general hospital of Al Wakra. Regarding residential care, this study found three new services: a women’s wellness inpatient unit delivering acute non-hospital care (R0) with 12 beds, and a new subacute unit for long-stays in the Rumailah hospital (R4) replacing the previous one (18 beds), and an acute unit in a general hospital (R2) in Al Wakra (12 beds). Finally, a 24-h hotline offered information and guidance for mental health issues through a specialized staff (I2.1.2).

Therefore, the availability of specialized services increased in this period for acute hospital care (0.13 per 100,000 inhabitants), and non-acute health-related outpatient care (0.13 for mobile care and 0.31 for non-mobile care). Moreover, the care pattern was expanded with the acute non-hospital residential care (0.04 per 100,000 inhabitants). The service availability for children and adolescents and older adults remained similar. Since 2018, the weight of the non-health related services or balance of care decreased (7.4%), while the diversity of care (10 codes) slightly increased.

## 4 Discussion

This study is the first standard description and classification of mental health services carried out in Qatar and, by extension, in a country of the Persian Gulf in the Eastern Mediterranean. This region joins other world areas where a comprehensive study on psychiatric service provision was previously done in the framework of the Glocal project (Global and Local Observation and mapping of CAre Levels) (20): Europe (21–24), South West Pacific (25–29), and South America (30), as well as other areas where a standard description and classification of services has supported other types of service research, such as Africa and North America (15).

The healthcare ecosystem research approach followed in this study has a bottom-up perspective to provide local scientific evidence for service planning. This information can be combined with other sources, such as service use and population health datasets, as well as top-down studies as the WHO’s Mental Health Atlas (11). The results can also be analyzed by using modeling techniques to produce new information, such as data envelopment analysis (31–33) and artificial neuronal networks (34).

This study provides the analysis of the mental health service provision in the time span of two national mental health plans, and a national health strategy. Therefore, it was an opportunity to evaluate the implementation of mental healthcare plan in Qatar. Moreover, it is worth mentioning that this research is the product of the collaboration between local and international researchers. The promotion of international collaborations is considered essential to improve mental health research in Arab countries (35).

The first mental health strategy (1) aimed at the creation of comprehensive and integral services in the system. External WHO experts, who assessed the impact of the strategy after the first year, stated that the main challenge was overcoming the historical situation of the Qatari mental health system (36). According to this assessment, most mental healthcare was concentrated in a monographic hospital in Doha with a stigmatizing image, there was no integration of mental healthcare into general hospitals, and specialized mental health services were not organized in catchment areas.

The external assessment found seven mental health outpatient services, one mental hospital, 2 day-treatment facilities, and one community residence in 2014. This study has identified 12 outpatient services (including liaison services, outreach services, and outpatient clinics), and two community residences in 2018. comparing our availability rates with those calculated in this report, at the end of the strategy time span outpatient services increased from 0.31 to 0.35 per 100,000 inhabitants from 2014 to 2018; day services stayed the same; community residences from 0.04 to 0.09, and inpatient hospital units from 0.04 to 0.13. The intense increase of the population in Qatar should be considered in the comparison of these rates. The strategy proposed to include 50% of psychiatric beds in general hospitals by 2018, but it has not yet been fulfilled since only the Al Wakra general hospital has an acute unit. Children and adolescent services had a major launch with the incorporation of Sidra Medicine services for children and perinatal health. Although the advance was quick and great, the former Executive Director of the National Mental Health Program stated that there was still a long way to go (37).

The second strategic plan (2) sought to provide high quality, integrated mental health services both in the community and across inpatient mental health services. Specifically, the strategy developed a range of new community settings, the inclusion of non-clinic professionals, and the provision of mental healthcare in primary care to change the role of the previous psychiatric facilities. This strategy assessed the achievements of the previous strategy as positive in the development of community services for specific populations. However, the new plan did not address in its priorities the organization of services in catchment areas as raised by the external assessment report (36).

According to our study, in the framework of this second strategy new services for young adult mental health were created linked to Qatar University, and three services started up outside of Doha. A new experimental residential service was available for women too (38). Other action was the reorganization of the subacute unit for males that dramatically reduced its number of beds in the last 5 years. However, the services for children and adolescents and older adults remained similar. There was also a specific service aimed at migrant workers that reach this population at risk, often far from the health system (39). To our knowledge, there has not been any published assessment of this strategy.

The National Health Strategy 2018–2022 (3) diagnosed that the Qatari mental health system had a limited availability of services, especially of outpatient facilities, and beds in community residences and psychiatric hospitals. The plan aimed to develop mental health in primary care and mental health support clinics. The strategy also pointed out a national target to achieve 20% of care delivered in the primary and community sectors. Indeed, our study showed an extraordinary increment of outpatient and community resources between 2018 and 2022.

The results showed that the Qatari mental health system followed a community model that progressed to be less hospital-based. In 5 years, the provision increased in eight new services providing a wide range of types of care such as information for care, outpatient, and residential care. Moreover, this improvement was produced in a world crisis period where the priority of governments was dealing with the COVID-19 pandemic, and its consequent economic downturn. Qatar was one of the countries with the highest positive cases in the first months of the pandemic, affecting mainly immigrants. This caused the increase in demand for mental health services (40).

The comparison of Qatar with European and Australian health areas in the Glocal project dataset (20) indicates that Qatari care pattern was much closer to the European regarding the absence or weakness of social outpatient care and accessibility to care (22, 41), while the lower variability in day care was more similar to the Australian pattern (26, 29).

This study presents several limitations. Even though DESDE-LTC has proven useful to estimate workforce capacity (27), our study did not focus on this aspect of healthcare. Furthermore, the lack of similar mental health service provision studies in the Middle East impeded the comparison of the Qatari care pattern with other countries of the EMRO Region.

## 5 Conclusion

This study examined the evolution of mental health services in Qatar (2018–2022) in the framework of a specific mental health and a general health plan. We used an international validated and widely applied methodology to assess and describe mental health services (DESDE-LTC). This is the first time that such methodology is applied in a Middle East country to study its mental healthcare pattern. In this period, the Qatari mental health service provision increased for adult population, while for other age groups remained the same. It was noteworthy the development of new services for young adults, migrant workers, and female populations. Our results showed the last advances to consolidate and widen the integrated model with the quick creation of new services and transformation of some of the previous ones fulfilling the objectives of the health planning.

## Data availability statement

The raw data supporting the conclusions of this article will be made available by the authors, without undue reservation.

## Author contributions

LS-C, MG-C, and NB designed the study. NB, SG, and JS-P collected the data. MG-C and LS-C classified the service with DESDE-LTC instrument. JS-P carried out the quantitative analysis and visualizations. SG and JS-P wrote the working draft. All authors reviewed and accepted the manuscript.
